# Supplementation with Rare Earth–Chitosan Chelate Improves Tibia Quality, Disease Resistance Capacity, and Performance in Nursery Pigs

**DOI:** 10.3390/ijms26062409

**Published:** 2025-03-07

**Authors:** Shaobin Hao, Wenchen Sun, Panting Wei, Huadong Wu, Wei Lu, Yuyong He

**Affiliations:** 1 Jiangxi Province Key Laboratory of Animal Nutrition and Feed, Engineering Research Center of Feed Development, Jiangxi Agricultural University, Nanchang 330045, China; haoshaobin1775@163.com (S.H.); sun1221_wc@163.com (W.S.); 18679954991@163.com (P.W.); lw20030508@163.com (W.L.); 2 College of Animal Science and Technology, Jiangxi Agricultural University, Nanchang 330045, China; whd0618@163.com

**Keywords:** fecal microbiota, liver, immune, inflammation, nursery pig, rare earth–chitosan chelates

## Abstract

The aim of this study was to investigate the effects on the tibia, liver, and gut, and on performance, when supplementing nursery pigs with different levels of rare earth–chitosan chelate (RECC). A total of 80 piglets, weaned at 7.67 ± 0.09 kg, were randomly assigned to groups RECC0 (RECC, 0 mg/kg diet), RECC200 (RECC, 200 mg/kg diet), RECC400 (RECC, 400 mg/kg diet), and RECC600 (RECC, 600 mg/kg diet), with four replicates in each group and five pigs per replicate during a 28 d experiment. Samples of the left hind tibia, serum, and feces were collected for analysis. The results indicated that, compared to pigs from group RECC0, pigs from group RECC200 presented with the following: a longer trabecular perimeter (*p* < 0.05), a larger trabecular area (*p* < 0.01), a higher trabecular number (*p* < 0.05), a smaller degree of trabecular separation (*p* < 0.01), and a lower number of osteoclasts (*p* < 0.01) in the tibia; higher abundances of beneficial fecal bacteria such as *g_Prevotellaceae_NK3B31_group*, *g_UCG_005*, *g_Rikenellaceae_RC9_gut_group*, *g_Acetitomaculum*, *g_Glutamicibacter*, *g_Frisingicoccus*, and *g_Alistipes*; higher (*p* < 0.01) serum levels of IgM, IgA, IgG, and IL-10; a lower (*p* < 0.01) serum concentration of TNF-α; a higher (*p* < 0.05) average daily gain and feed conversion ratio; and a lower (*p* < 0.01) incidence of diarrhea. The dietary addition of RECC contributes to improvements in tibia quality, gut health, and performance in nursery pigs.

## 1. Introduction

In recent years, the incidence of limb bone diseases in intensive pig farms has been increasing, and the clinical symptoms mainly include lameness, instability, or paralysis [1]. It has been reported that osteomalacia mainly manifests as abnormal walking, decreased weight-bearing capacity, skeletal deformation, or pain due to insufficient bone mineralization [2]. Osteoporosis is a bone metabolism disorder characterized by decreased bone mass, imbalanced bone tissue absorption and formation, abnormal bone trabeculae, increased bone fragility, and decreased weight-bearing capacity, making bones prone to fractures [3].

A number of factors influence bone health, performance, and disease resistance in animals [4,5,6], and nutrition can influence these parameters in pigs by regulating the absorption of dietary nutrients and the composition of the gut microbiota [7,8,9]. Low dietary Ca intake results in bone loss by increasing vitamin D receptor expression in mice osteoclasts [10] or affecting mesenchymal stem cell activity in piglets [11]. The administration of phytase to gilts and barrows significantly reduces the cortical wall thickness and index of metacarpal bones by affecting the intake of calcium and phosphorus [12]. Supplementing growing gilts with copper, manganese, and zinc enhances the strength and density of bone [13].

Rare earth elements, especially lanthanum and cerium, are also used as safe feed additives to promote animal health, performance, and bone quality [14]. Bone formation is a complex process that occurs through the regulation of osteogenesis, osteoclastogenesis, and angiogenesis by nutrients [15]. Trivalent lanthanum can increase cortical bone density and bone volume, prevent the differentiation and maturation of osteoclasts, directly stimulate bone formation, and inhibit osteolysis by orchestrating the balance between osteoblasts and osteoclasts [16,17]. Local bone angiogenesis can accelerate bone development and fracture healing [18,19], and treating bone marrow mesenchymal stem cells with lanthanum or cerium ions was found to significantly promote angiogenesis [20]. Moreover, the addition of CeO to the diet of broilers improved tibia quality by increasing bone strength and osteocalcin gene expression [21].

The gut microbiota can also influence bone health [22,23,24] because gut dysbiosis can lead to bone diseases due to abnormal nutrient absorption and systemic inflammation [25,26,27]. It has been reported that chitosan can also improve animal health and performance, modulate gut microbiota [28], and induce bone formation [29]. Rare earth–chitosan chelates (RECC) are feed additives produced by chelating lanthanum and cerium ions from rare earth elements with chitosan. Previous experiments indicated that the dietary inclusion of RECC in laying hens at 100 mg/kg reduced the levels of interleukin 2 (IL-2) and tumor necrosis factor-alpha (TNF-α) [30]. In addition, the supplementation of RECC in the diet of sows during the perinatal period increased the relative abundances of *Ruminococcaceae* and *Christensenellaceae* but inhibited the growth of pathogenic *Proteobacteria* and *Campylobacter* and elevated serum levels of the growth hormone and insulin-like growth factor-1 [31].

The tibia is one of the most important bones in pigs, playing a crucial role in weight-bearing and walking [32]. However, the effects of RECC on the bone quality of nursery pigs have not been reported to date. The aim of this study was to investigate the effects of RECC on tibia quality, fecal microbiota, performance, immunity, and inflammation in nursery pigs, providing technical support for RECC addition into the diet of nursery pigs.

## 2. Results

### 2.1. Effects of Dietary RECC Addition on Performance of Nursery Pigs

The results in Table 1 show that nursery pigs from group RECC200 had a higher final live bodyweight (*p* < 0.01) and a higher average daily gain (*p* < 0.05) than nursery pigs from groups RECC0, RECC400, and RECC600, respectively. Meanwhile, nursery pigs from group RECC200 also had a lower feed-to-gain ratio (*p* < 0.05) and a lower incidence of diarrhea (*p* < 0.01) than nursery pigs from groups RECC0 and RECC600, respectively. As the levels of RECC increased, the final live body weight and average daily gain decreased, but the feed-to-gain ratio and the incidence of diarrhea increased, respectively.

### 2.2. Effects of Dietary RECC Addition on Tibia Microarchitecture of Nursery Pigs

**Figure 1:**
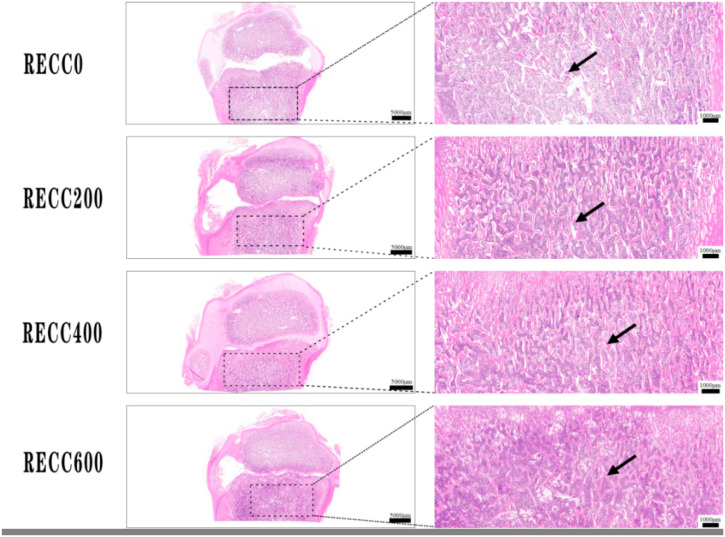
HE staining of trabecular bone in proximal tibia. Red irregular tissues indicated with black arrow.

**Figure 2:**
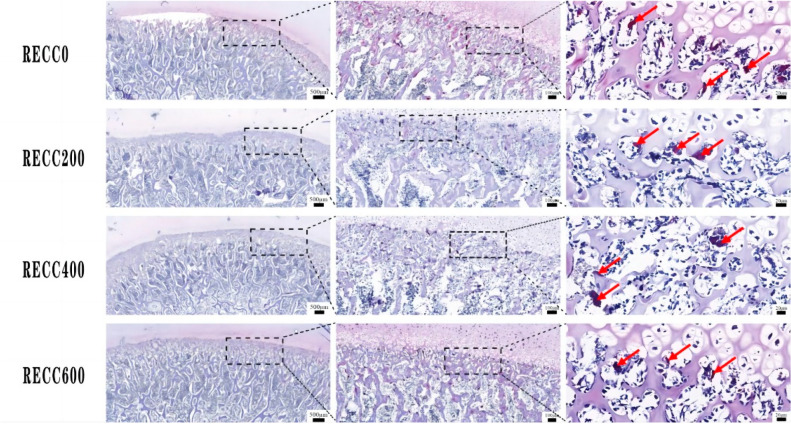
TRAP staining of osteoclasts in proximal tibia. TRAP-positive cells (dark red) indicated with red arrow.

The dietary addition of RECC to nursery pigs improved the trabecular bone microarchitecture (Figure 1) and decreased the number of osteoclasts (Figure 2). The results for the tibia microarchitecture, shown in Table 2, indicated that the dietary addition of RECC increased the trabecular perimeter, trabecular area, and trabecular number, but decreased the trabecular separation and osteoclast numbers. Groups RECC200 and RECC400 presented a longer trabecular perimeter (*p* < 0.05), a larger trabecular area (*p* < 0.01), a higher trabecular number (*p* < 0.05), a smaller degree of trabecular separation (*p* < 0.01), and a lower number of osteoclasts (*p* < 0.01) than group RECC0, respectively.

### 2.3. Effects of Dietary RECC Addition on Fecal Bacterial Composition of Nursery Pigs

**Figure 3:**
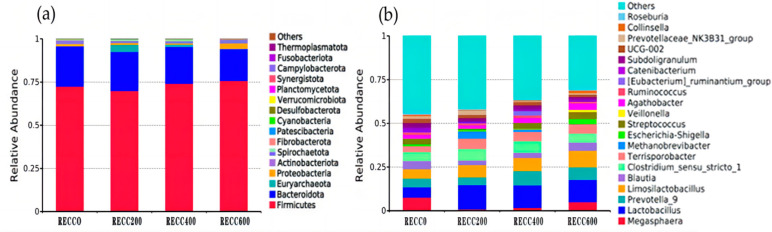
The composition of fecal bacteria at (**a**) the phylum level and (**b**) the genus level. The color-coded bar plot shows the average bacterial distribution in each group.

**Figure 4:**
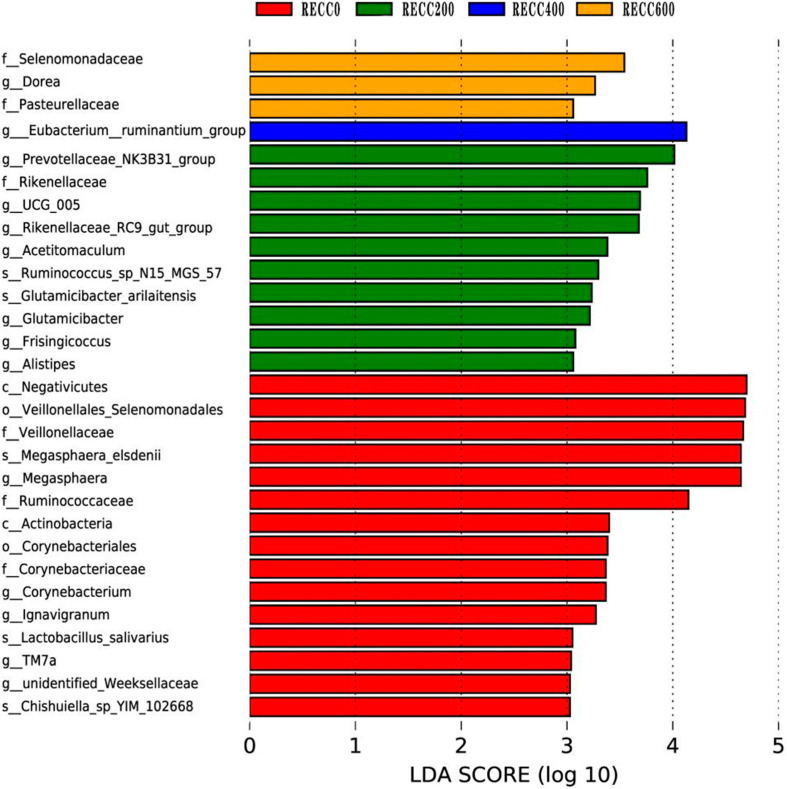
The differential bacteria among groups based on linear discriminant analysis (LDA) Effect Size (LEfSe). Each plot shows taxa with significant differences in abundance among groups, with the LDA score (log 10) indicating the effect size. Red, green, blue, and orange bars represent a higher abundance of bacteria at different taxonomic levels in groups RECC0, RECC200, RECC400, and RECC600, respectively.

The composition of fecal bacteria at the phylum and genus levels is presented in Figure 3. Firmicutes and Bacteroidota were the dominant phyla in the fecal samples of the four groups, but group RECC200 had a higher relative abundance of Euryarchaeota than the other three groups (Figure 3a). Dietary RECC addition increased the relative abundance of *Lactobacillus* but decreased the relative abundance of *Megasphaera* (Figure 3b). The alpha diversity results are listed in Table 3; the data indicated that groups RECC400 and RECC600 had lower indices of Ace, Chao 1, and Shannon than groups RECC0 and RECC200 (*p* < 0.05), respectively. There was no significant difference between groups RECC0 and RECC200 in terms of the alpha diversity of fecal bacteria (*p* > 0.05). Linear discriminant analysis Effect Size (LEfSe) analysis (LDA score ≥ 3) was used to identify the characteristic bacterial taxa of each group (Figure 4), and the results showed that the numbers of significantly different microbiota in groups RECC0, RECC200, RECC400, and RECC600 were 15, 10, 1, and 3, respectively. At the genus level, the characteristic bacteria were *g_Megasphaera*, *g_Corynebacterium*, *g_Ignavigranum*, *g_TM7a*, and *g_Unidentified_Weeksellaceae* in group RECC0; *g_Prevotellaceae_NK3B31_group*, *g_UCG_005*, *g_Rikenellaceae_RC9_gut_group*, *g_Acetitomaculum*, *g_Glutamicibacter*, *g_Frisingicoccus*, and *g_Alistipes* in group RECC200; *g_Eubacterium_ruminantium_group* in group RECC400; and *g_Dorea* in group RECC600.

### 2.4. Effects of Dietary RECC Addition on Hepatic Histomorphology of Nursery Pigs

**Figure 5:**
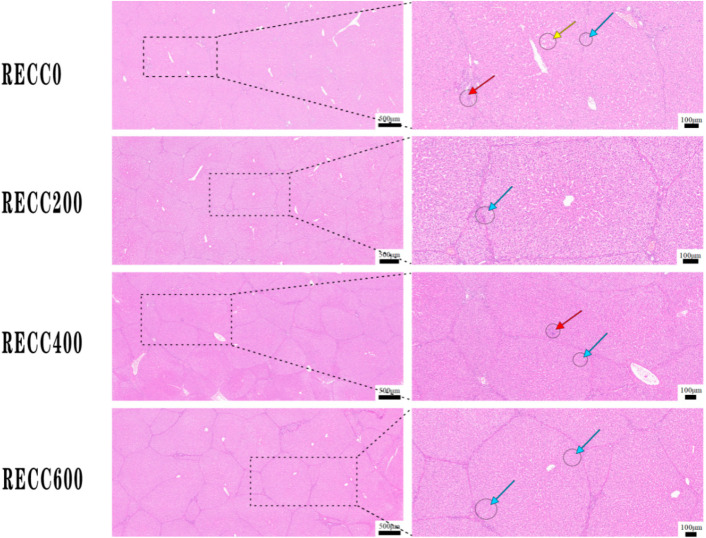
Alterations in histomorphology in livers of nursery pigs supplemented with RECC. Red arrow: liver lymphocytic infiltration, blue arrow: hepatocyte hydropic degeneration, yellow arrow: hepatic sinusoidal dilatation.

Figure 5 indicates that the nursery pigs from group RECC200 had less liver damage (lymphocytic infiltration, hepatocyte hydropic degeneration, or hepatic sinusoidal dilatation) than those from group RECC0.

### 2.5. Effects of Dietary RECC Addition on Jejunal Histomorphology of Nursery Pigs

The data in Table 4 indicate that the nursery pigs from group RECC200 had longer (*p* < 0.01) jejunal villi than those from groups RECC0, RECC400, and RECC600, respectively. The nursery pigs from groups RECC200, RECC400, and RECC600 had deeper (*p* < 0.01) crypts than those from group RECC0. The villus height/crypt depth ratio in the jejunum significantly increased (*p* < 0.05) when comparing nursery pigs from group RECC200 to nursery pigs from group RECC400.

### 2.6. Effects of Dietary RECC Addition on Immunoglobins and Cytokines in Serum of Nursery Pigs

The supplementation of RECC to nursery pigs increased the serum levels of IgM, IgA, IgG, and IL-10 and decreased the serum concentrations of IL-2 and TNF-α (Table 5). Compared to the nursery pigs from groups RECC0 and RECC600, those from groups RECC200 and RECC400 had higher (*p* < 0.01) levels of IgM and IgA in serum, respectively. The nursery pigs from groups RECC200, RECC400, and RECC600 had higher (*p* < 0.01) levels of IgG and IL-10 in serum than the pigs from group RECC0, respectively. The level of TNF-α was lower (*p* < 0.01) in the serum of nursery pigs from group RECC200 than in that of the pigs from groups RECC0, RECC400, and RECC600, respectively.

## 3. Discussion

The parameters (trabecular number, thickness, and spacing) of the trabecular bone and the number of osteoclasts are often used to assess bone quality in animals [33]. Increased trabecular spacing results in the loss of bone density [34,35]. Osteoclasts are a kind of mobile cell with multiple nuclei that attach to the bone surface to break down and absorb the organic and inorganic components of bones; an increase in the number of osteoclasts can lead to the deterioration of bone density [36]. The results of this study showed that the dietary addition of RECC reduced the number of osteoclasts in the tibia of nursery pigs. It is well known that many factors can exert influences on bone quality in animals, and many researchers are currently examining the effects of rare earth elements, rare earth–chitosan chelate, and the gut microbiota on the bone quality of commercial and breeding animals. Prior studies have reported that cerium ions can increase the trabecular thickness and decrease the trabecular spacing of rat skulls [20], and the addition of La(NO_3_)3 to bone marrow mesenchymal stem cells from the tibia of mice was found to significantly increase trabecular thickness compared to the untreated group [37]. Lanthanum trivalent ions can inhibit the formation of osteoclasts [17,37,38]. In this study, the dietary addition of RECC at 200 or 400 mg/kg to the diet of nursery pigs significantly increased the trabecular number and area and decreased the trabecular spacing and number of osteoclasts in the tibia. Bone loss can be reduced by inhibiting osteoclastogenesis through the blocking of IL-21 expression [37]. The results of this study also indicated that dietary RECC supplementation reduced the number of osteoclasts in the tibia, decreased serum TNF-α levels, and increased serum IL-10 levels. It has been reported that decreased TNF-α levels and increased IL-10 levels may improve bone quality by suppressing osteoclast formation [39,40]; one possible pathway through which IL-10 suppresses osteoclast formation may be through the regulation of E2 on bone metabolism by enhancing regulatory T cell production. Bone development and health are also affected by the gut microbiota [41,42]. Some *Lactobacillus* spp., including *L. reuteri*, *L. rhamnosus*, and *L. paracasei*, can prevent bone loss by decreasing osteoclastogenesis and bone resorption in mouse models [43,44]. Oral CeO_2_ administration significantly decreased the abundance of *Lactobacillus* in the gut of rats [45,46], but dietary lanthanum and cerium supplementation increased fecal *Lactobacillus* in pigs [47]. Short-chain fatty-acid-producing bacteria play vital roles in reducing bone loss and improving bone mineral density [48]. Among these, Alistipes are usually considered one of the protective factors for bone mineral density [48] as they increase mineral absorption [49,50]. Previous results are further validated by the data obtained in this study because the nursery pigs supplemented with RECC had better tibia quality and higher relative abundances of *g_Lactobacillus* and *g_Alistipes*.

The gut microbiota can not only affect the quality of bones but is also involved in disease resistance and performance in animals [51]. Previous studies reported that *Citrobacter rodentium* infection can induce colitis, and *g_Prevotellaceae-NK3B31-group* can reduce infection by inhibiting the growth of *Citrobacter rodentium* [52]. The reduction in gut inflammation results in the improvement of disease resistance capacity and performance, a process that is further verified by the results of this study. Feeding *g_Rikenellaceae_RC9_gut_group* to pigs can increase the feed conversion ratio and reduce backfat thickness [53]. *g_Acetitomaculum* often generate volatile fatty acids by fermenting glucose and other carbohydrates [54,55,56] and show a strong positive correlation with the levels of IL-10 [57]. *g_Alistipes* are short-chain fatty-acid-producing bacteria [58,59] that can decrease the inflammatory response and liver diseases [49,50,60]. *g_Eubacterium_ruminantium_group* can reduce inflammation by producing short-chain fatty acids [61,62,63], improving gut histomorphology [64]. *g_Dorea* can exert anti-inflammatory effects by producing short-chain fatty acids (acetate, propionate, and butyrate) through the fermentation of starch, cellulose, and hemicellulose in the gut [65,66,67]. In addition, *g_Dorea* have positive genetic correlations with backfat thickness [53,68]; backfat thickness can affect both the value of the carcass and the reproductive traits of sows [69], so the genetic control of *g_Dorea* has major economic importance for breeding sows. The results of this study indicated that the nursery pigs treated with a 200 mg/kg diet of RECC had higher relative abundances of *g_Prevotellaceae_NK3B31_group*, *g_Rikenellaceae_RC9_gut_group*, *g_Alistipes*, and *g_Acetitomaculum* than those without RECC treatment, contributing to the improved performance and disease resistance outcomes among nursery pigs. The data obtained in this study also indicated that the nursery pigs from group RECC0 had high levels of IL-2 and TNF-α, high diarrhea incidence, a poor feed conversion ratio, and low levels of IL-10, which might be attributed to the high fecal numbers of *g_Megasphaera*, *g_Corynebacterium*, *g_Ignavigranum*, and *g_TM7.* It is reported that *g_Megasphaera* can reduce the feed conversion ratio [70]. *g_Corynebacterium* has a positive correlation with pro-inflammatory cytokines and ferroptosis, but a negative correlation with IL-10 [71]. *g_Ignavigranum* are inflammation-producing bacteria identified in the milk of cows infected with mastitis [72]. *g_TM7* are also associated with inflammatory bowel diseases, which are characterized by high diarrhea incidence and pro-inflammatory cytokines [73].

It is well known that increasing the content of IgA, IgG, and IgM can improve disease resistance in animals. The addition of dietary chitosan increased the content of IgA, IgG, and IgM in the serum of Huoyan geese [74]. In this study, the data demonstrated that dietary RECC addition also increased the concentration of IgA, IgG, and IgM in the serum of nursery pigs. The morphological and functional integrity of the gut mucosa is important for nutrient absorption and the performance of livestock [75]. The addition of rare-earth-containing lanthanum and cerium to the diet at levels from 0 to 600 mg/kg had no significant impact on the jejunal histomorphology of hens during the late laying stage [76]. However, the addition of dietary lanthanum significantly improved the intestinal morphology of finishing pigs [44], and the addition of a rare earth element (Azomite) to the diet at 2.5–5.0 g/kg also significantly elevated the intestinal villus height of tilapia [77]. The results of this study indicated that supplementing nursery pigs with RECC at 200 mg/kg diet significantly improved jejunal histomorphology; this is possibly attributed to decreased inflammation in the gut.

## 4. Materials and Methods

### 4.1. Materials

Chitosan is a naturally occurring linear polysaccharide of glucosamine and N-acetylglucosamine that is obtained through the deacetylation of chitin. It exhibits stable coordination with rare earth ions through hydrogen or salt bonds due to the presence of numerous hydroxyl, amino, and N-acetylamino groups in the chitosan molecular chain. RECC is a novel feed additive formed via coordination chelation between rare earth ions and the amino and hydroxyl groups on chitosan molecules through electrochemical processes.

The RECC product used in this study was provided by Jiangxi Weiting Industrial Co., Ltd. (Jiangxi, China). It contains 30% RECC and 70% carriers (chitosan, montmorillonite, and calcium carbonate), and the concentrations of lanthanum and cerium in this RECC product are 5.12% and 3.26%, respectively.

### 4.2. Animal Experimental Design

A total of 80 crossbred piglets (Duroc × Landrace × Yorkshire) weaned at 26 days with an average bodyweight of 7.67 ± 0.09 kg were randomly assigned to group RECC0 (RECC, 0 mg/kg diet), group RECC200 (RECC, 200 mg/kg diet), group RECC400 (RECC, 400 mg/kg diet), or group RECC600 (RECC, 600 mg/kg diet). Each group included 20 piglets (10♂, 10♀) with four replicates (pens), and each pen had five piglets.

### 4.3. Feed Preparation and Animal Feeding

The commercial diet for the nursery pigs was purchased from a feed factory. The nutrient levels of this commercial diet were analyzed and are listed as follows (on a dry matter basis): dry matter, 89.10%; crude protein, 17.40%; ether extract, 4.70%; crude fiber, 1.80%; crude ash, 6.00%; calcium, 0.75%; and total phosphorus, 0.68%. The test feed offered to the nursery pigs in each group was prepared as follows. First, the daily requirements for the RECC product and commercial diet for nursery pigs in each treatment group were calculated and weighed. Second, the RECC product was dissolved in tap water at a ratio of 1:100 (weight/weight) and was then mixed well with the feed in a small feed mixer.

All piglets were kept in concrete-floored pens. The room temperature was set at 28 °C for the first week and then gradually reduced by 1 °C to 25 °C for the fourth week. The humidity in the room was 63.6%, and ventilation was provided by an exhaust fan with a diameter of 70 cm. The test feed was artificially placed into the feed trough, and all nursery piglets had free access to the test feed and water throughout the 28-day experiment.

### 4.4. Data Collection and Calculation

All piglets were weighed at the beginning and the end of experiment, respectively, and the average daily gain (ADG) was calculated as follows: ADG (g/d) = (final bodyweight − initial bodyweight)/experimental days. The feed offered and refused were also recorded each day, and the average daily feed intake (ADFI) was calculated with the formula ADFI (g/d) = (feed offered − feed refusal)/experimental days. The number of piglets with diarrhea symptoms was recorded every day, and the diarrhea incidence (%) was calculated as ([number of piglets with diarrhea within a treatment]/[number of piglets × total experimental days]) × 100, where the “number of piglets with diarrhea” was the total number of piglets with diarrhea observed each day [78].

### 4.5. Sample Collection and Preparation

Fecal samples were collected from the anus of each nursery pig on days 13 and 26, respectively. After the collection at each time point, fecal samples from the same treatment group were mixed, placed into sterile plastic tubes with 3 replicates, and stored in liquid nitrogen for 16S rDNA sequencing.

After 12 h of fasting, a total of eight piglets were randomly selected from each group (1♂ and 1♀ per pen) on day 28 to collect blood samples from the precaval vein into nonheparinized vacuum tubes, and after standing for one hour at room temperature (22 °C), all blood samples were centrifuged at 3000× *g* for 10 min at 4 °C. The supernatant of each vacuum tube was pipetted into new sterile EP tubes as aliquots, and the serum samples were stored at −20 °C until analysis.

At the end of the experiment, a total of 4 piglets (2♂, 2♀) with approximately average body weights were selected from each group (1 piglet/pen) and bled after electrical stunning. After opening the abdominal cavity, the liver and intestine were taken out. The liver samples were collected from the inferior margin of the right lobe, and jejunal segments of approximately 2 cm in length were removed from the middle of the jejunum and flushed with normal saline until the digesta was thoroughly removed. The left proximal tibia was harvested and the upper part of the tibia was halved longitudinally in two parts with a saw. The samples of liver, jejunum, and tibia were then immediately fixed with 4% paraformaldehyde solution for 24 h. After 24 h fixation, the tibia samples were decalcified in a 10% ethylenediaminetetraacetic acid (EDTA) solution at a pH of 7.4 at room temperature for 5 weeks.

### 4.6. Tissue Staining

The treated tibia, liver, and jejunum samples were then dehydrated in ethanol solution with different concentrations, embedded in paraffin, and longitudinally cut into sections with a thickness of 5 μm. After deparaffinization and rehydration, sections of the liver, jejunum, and tibia were subjected to staining with hematoxylin and eosin (HE) kits (Solarbio, Beijing, China) according to the manufacturer’s protocols. In addition, the tibia sections were also stained with tartrate-resistant acid phosphatase (TRAP) kits (Sigma-Aldrich, St. Louis, MO, USA) following the manufacturer’s instructions to detect the osteoclasts. TRAP-positive multinucleated cells were considered to be osteoclasts [37].

After staining, the HE- and TRAP-stained slides were scanned using a Pannoramic DESK/MIDI/250/1000 digital scanner (3DHISTECH Ltd., Budapest, Hungary). The target images were captured with Case Viewer software 2.2 (3DHISTECH Ltd., Budapest, Hungary). The Image-Pro Plus 6.0 software (Media Cybernetics, Bethesda, MD, USA) was used to measure the villus height and crypt depth (randomly measuring 5 villi and 5 crypts per section) of the jejunum, the histomorphometry parameters, and the osteoclast numbers of the tibia, respectively.

### 4.7. Fecal Bacteria Sequencing

The DNA of fecal bacteria obtained from each sample was extracted using DNeasy PowerSoil Kits (Qiagen, Hilden, Germany) according to the manufacturer’s protocols. The qualified DNA samples were amplified by targeting the V3-V4 region using the primers 343F (5′-TACGGRAGGCAGCAG-3′) and 798R (5′-AGGGTATCTAATCCT-3′). PCR reactions were performed in a mixture containing 15 µL of Phusion ➅ High-Fidelity PCR Master Mix, 0.2 µM of the primers, and 10 ng of the template DNA with the following conditions: initial denaturation at 98 °C for 1 min, followed by 30 cycles of denaturation at 98 °C for 10 s, annealing at 50 °C for 30 s, elongation at 72 °C for 30 s, and finally, 72 °C for 5 min.

The PCR products were extracted from 2% agarose gels and purified using Vazyme DNA clean beads. After purification, the PCR products were quantified with a Qubit 3.0 Fluorometer (Invitrogen, Waltham, MA, USA) to generate amplicon libraries. The libraries were pooled in equimolar amounts and paired-end-sequenced (PE250) at Novogene Co., Ltd. (Beijing, China) on an Illumina NovaSeq 6000 platform (Illumina, San Diego, CA, USA). Bioinformatics analysis was performed according to a previously published method [79]. The raw sequencing data were deposited in the Sequence Read Archive of the National Center for Biotechnology Information under the BioProject ID PRJNA1056486.

### 4.8. Serum Sample Analysis

The concentrations of immunoglobin (Ig)A, IgG, IgM, interleukin (IL)-10, IL-2, and tumor necrosis factor-alpha (TNF-α) of the serum samples were tested using enzyme-linked immunosorbent assay kits purchased from Shanghai Enzyme-linked Biotechnology Co., Ltd. (Shanghai, China).

### 4.9. Statistical Analysis

The data were analyzed using the Prism Software 5.0 (GraphPad Software, Inc., San Diego, CA, USA), and normal distribution was determined using the Shapiro–Wilk test. The statistical significance (*p* < 0.05) was evaluated via one-way ANOVA and Tukey’s multiple comparisons, and the results are presented as means with the standard error of the mean.

## 5. Conclusions

Supplementing RECC into the diet at a level of 200 mg/kg contributes to a higher trabecular perimeter, trabecular area, and trabecular number; a lower degree of trabecular separation and a lower osteoclast number; and higher abundances of the beneficial bacteria *Prevotellaceae_NK3B31_group*, *UCG_005*, *Rikenellaceae_RC9_gut_group*, *Acetitomaculum*, *Glutamicibacter*, *Frisingicoccus*, and *Alistipes*, thus improving the disease resistance capacity and performance of nursery pigs. The findings of this study provide novel insights into improving bone quality, health, and performance in nursery pigs via the administration of RECC to alter the gut microbiota; however, the underlying mechanisms remain unclear and should be explored further in subsequent studies.

## Figures and Tables

**Table 1:** Performance of nursery pigs supplemented with different RECC levels.

	RECC0	RECC200	RECC400	RECC600
Initial live body weight (kg)	7.53 ± 0.05	7.67 ± 0.10	7.78 ± 0.09	7.71 ± 0.07
Final live body weight (kg)	18.59 ± 0.09 ^C^	20.17 ± 0.02 ^A^	19.59 ± 0.08 ^B^	19.47 ± 0.10 ^B^
Average daily gain (g/d)	395.14 ± 4.78 ^c^	446.25 ± 3.04 ^a^	421.93 ± 0.22 ^b^	420.12 ± 1.19 ^b^
Average daily feed intake (g/d)	645.45 ± 0.74	669.94 ± 4.61	678.18 ± 12.01	693.29 ± 13.39
Feed intake/body weight gain	1.63 ± 0.02 ^a^	1.50 ± 0.01 ^b^	1.61 ± 0.03 ^ab^	1.65 ± 0.05 ^a^
Diarrhea incidence (%)	18.39 ± 1.61 ^Aa^	5.89 ± 1.25 ^Bb^	9.46 ± 1.25 ^ABb^	15.00 ± 1.43 ^Aa^

Data are presented as the “Mean ± SEM”. Mean values within a row differ significantly at *p* < 0.01 with different capital letters, or at *p* < 0.05 with different lowercase letters.

**Table 2:** Effects of RECC supplementation on trabecular bone and osteoclasts in tibia.

	RECC0	RECC200	RECC400	RECC600
Tb.Pm (mm)	522.90 ± 60.23 ^b^	731.88 ± 61.39 ^a^	726.55 ± 44.34 ^a^	645.41 ± 41.14 ^ab^
Tb.Ar (mm^2^)	7.01 ± 0.54 ^Cc^	9.50 ± 0.69 ^ABb^	11.31 ± 0.62 ^Aa^	7.92 ± 0.46 ^BCbc^
Tb.Th (um)	24.79 ± 2.13 ^ab^	22.22 ± 1.35 ^ab^	26.36 ± 1.11 ^a^	21.18 ± 1.44 ^b^
Tb.N (1/mm)	6.42 ± 0.74 ^b^	8.99 ± 0.75 ^a^	8.92 ± 0.54 ^a^	7.93 ± 0.51 ^ab^
Tb.Sp (um)	167.14 ± 28.07 ^Aa^	97.84 ± 8.36 ^Bb^	91.88 ± 8.02 ^Bb^	112.83 ± 9.51 ^ABb^
N.Oc (N/mm^2^)	81.11 ± 9.93 ^A^	19.31 ± 3.36 ^B^	27.04 ± 3.84 ^B^	28.14 ± 5.93 ^B^

Tb.Pm: trabecular perimeter, Tb.Ar: trabecular area, Tb.Th: trabecular thickness, Tb.N: trabecular number, Tb.Sp: trabecular separation/spacing, N.Oc: number of osteoclasts. Data are presented as the “Mean ± SEM”. Mean values within a row differ significantly at *p* < 0.01 with different capital letters, or at *p* < 0.05 with different lowercase letters.

**Table 3:** Alpha diversity of fecal bacteria in different groups.

	RECC0	RECC200	RECC400	RECC600
Ace	649.25 ± 36.65 ^a^	654.37 ± 11.04 ^a^	550.39 ± 26.89 ^b^	571.06 ± 16.78 ^b^
Chao1	651.23 ± 33.69 ^a^	660.39 ± 10.06 ^a^	553.89 ± 28.63 ^b^	575.48 ± 17.54 ^b^
Shannon	6.35 ± 0.28 ^a^	6.17 ± 0.09 ^ab^	5.79 ± 0.16 ^b^	5.64 ± 0.09 ^b^
Simpson	0.96 ± 0.01	0.95 ± 0.01	0.95 ± 0.01	0.95 ± 0.01

Ace, abundance-based coverage estimator. Data are presented as the “Mean ± SEM”. The mean values within a row differ significantly at *p* < 0.05 with different lowercase letters.

**Table 4:** Jejunal histomorphology of nursery pigs supplemented with different RECC levels.

	RECC0	RECC200	RECC400	RECC600
Villus height (mm)	0.38 ± 0.02 ^B^	0.49 ± 0.03 ^A^	0.39 ± 0.01 ^B^	0.37 ± 0.02 ^B^
Crypt depth (mm)	0.28 ± 0.01 ^B^	0.35 ± 0.01 ^A^	0.33 ± 0.01 ^A^	0.34 ± 0.01 ^A^
Villus height/crypt depth	1.40 ± 0.09 ^Aa^	1.42 ± 0.09 ^Aa^	1.19 ± 0.04 ^ABb^	1.09 ± 0.04 ^Bb^

Data are presented as the “Mean ± SEM”. The mean values within a row differ significantly at *p* < 0.01 with different capital letters, or at *p* < 0.05 with different lowercase letters.

**Table 5:** Serum immunoglobins and cytokines of nursery pigs supplemented with different amounts of RECC.

	RECC0	RECC200	RECC400	RECC600
IgM (μg/mL)	104.51 ± 5.55 ^B^	145.98 ± 6.61 ^A^	146.81 ± 3.55 ^A^	106.24 ± 3.81 ^B^
IgA (μg/mL)	40.00 ± 1.98 ^C^	66.13 ± 1.68 ^A^	62.30 ± 2.04 ^A^	52.16 ± 2.46 ^B^
IgG (g/L)	14.07 ± 0.72 ^B^	21.59 ± 1.25 ^A^	22.21 ± 0.81 ^A^	19.76 ± 0.54 ^A^
IL-10 (pg/mL)	147.65 ± 3.29 ^Cb^	184.23 ± 4.07 ^ABa^	175.99 ± 6.43 ^Ba^	157.57 ± 6.98 ^BCb^
IL-2 (pg/mL)	282.07 ± 16.52	254.14 ± 11.43	273.08 ± 11.44	269.84 ± 11.28
TNF-α (pg/mL)	222.95 ± 5.95 ^Aa^	167.67 ± 10.83 ^Bc^	205.31 ± 5.71 ^Aab^	193.92 ± 7.75 ^Ab^

Data are presented as the “Mean ± SEM”. The mean values within a row differ significantly at *p* < 0.01 with different capital letters, or at *p* < 0.05 with different lowercase letters.

## Data Availability

The original contributions presented in the study are included in the article, and further inquiries can be directed to the corresponding author.

## Abbreviations

The following abbreviations are used in this manuscript:

RECC	Rare earth–chitosan chelate
IgM	Immunoglobulin M
IgA	Immunoglobulin A
IgG	Immunoglobulin G
IL-10	Interleukin-10
IL-2	Interleukin-2
TNF-α	Tumor necrosis factor-α
Tb.Pm	Trabecular perimeter
Tb.Ar	Trabecular bone area
Tb.Th	Trabecular thickness
Tb.N	Trabecular number
Tb.Sp	Trabecular separation
N.Oc	Osteoclast number
ADG	Average daily gain
ADFI	Average daily feed intake
